# Prospective associations of infant food exposures and appetitive traits with early childhood diet quality

**DOI:** 10.1186/s12966-024-01686-4

**Published:** 2024-12-18

**Authors:** Tonja R. Nansel, Amara Channell-Doig, Leah M. Lipsky, Kyle Burger, Grace Shearrer, Anna Maria Siega-Riz, Yong Ma

**Affiliations:** 1 Social and Behavioral Sciences Branch, Division of Population Health Research, Eunice Kennedy Shriver National Institute of Child Health and Human Development, 6710B Rockledge DrMSC 7004, Bethesda, MD 20892 USA; 2 Department of Nutrition, University of North Carolina, Chapel Hill, USA; 3 Department of Family and Consumer Sciences, University of Wyoming, 1000 University, Laramie, WY 82071 USA; 4 Epidemiology and Biostatistics, School of Public Health and Health Sciences, University of Massachusetts, 715 North Pleasant St., 109 Arnold, Amherst, MA 01003-9303 USA; 5 Glotech Group, Contractor for the Division of Population Health Research, Eunice Kennedy Shriver National Institute of Child Health and Human Development, 6710B Rockledge DrMSC 7004, Bethesda, MD 20892 USA

**Keywords:** Infant, Child, Feeding, Complimentary feeding, Fruits, Vegetables, Discretionary foods, Noncore foods, Diet quality, Appetitive traits

## Abstract

**Background:**

Early-life food exposures may influence food preferences and receptivity, thereby impacting long-term diet quality. Infant exposure to discretionary foods may be more detrimental for infants with high food approach traits; conversely, early exposure to fruits and vegetables may be more important for those with high food avoidance traits. This study investigated associations of infant food exposures with early childhood diet quality and whether these associations are modified by infant appetitive traits.

**Methods:**

Data are from the Pregnancy Eating Attributes Study (PEAS) and Sprouts follow-up study, a prospective cohort assessed from the first trimester of pregnancy through early childhood. Birthing parents completed the Baby Eating Behavior Questionnaire assessing food-approach and food-avoidance appetitive traits at 6 months and food frequency questionnaires assessing infant age at introduction to and frequency of consuming food groups at ages 6 months, 1 year, and 2 years. At child ages 3.5 and 5 years, parents provided two 24-h dietary recalls, from which the Healthy Eating Index-2020 was calculated to measure diet quality. Structural equation models using maximum likelihood estimation examined associations of age at introduction to, and intake frequency of, fruit/vegetables and discretionary foods, and their interactions with food-approach and food-avoidance appetitive traits, on child diet quality at ages 3.5 & 5 years, controlling for income, education, and breastfeeding duration.

**Results:**

Higher childhood diet quality was associated with later infant age at introduction to discretionary foods, lower intake of discretionary foods at ages 1 and 2 years, and greater intake of fruits and vegetables at age 2 years. Childhood diet quality was not associated with infant age at introduction to fruits and vegetables. Intake of fruits and vegetables at age 1 year interacted with food avoidance traits, indicating that the association of fruit and vegetable intake with childhood diet quality was magnified by greater food avoidance.

**Conclusions:**

Exposure to discretionary food in the first two years of life was consistently associated with lower diet quality in early childhood regardless of the strength of appetitive traits. Findings suggest that improving child diet quality may require stronger efforts to limit exposure to discretionary foods in infancy.

## Background

The impact of nutrition in the first thousand days on long-term health is recognized by medical and public health professionals [1–3]. Healthful dietary intake during this time promotes optimal physical development [4, 3]; low diet quality is associated with disease risk including poorer cardiometabolic health [5–7], increased childhood asthma [8, 9], food allergies [10–12], and increased adiposity [5, 13–15]. Beyond the physical impact on development, early dietary intake lays the foundation for long-term dietary patterns.

Early food exposures may impact children’s diet quality long-term by influencing food preferences and receptivity to healthful foods such as vegetables, fruit, and whole grains [16]. Conversely, early and frequent exposure to discretionary foods (e.g., desserts, salty snacks) may reduce motivation for and intake of healthful foods, while increasing expectations and requests for discretionary foods [17]. As such, intake patterns may track across development. Intake of vegetables, fruits, noncore (discretionary) foods, and sugar-sweetened beverages in infancy predicted intake of the corresponding food groups in early childhood [18–21]. Similarly, infant overall diet quality [22] and later introduction of sugar-sweetened beverages [23] were associated with greater HEI in early childhood.

The influence of early feeding exposures on subsequent diet quality may vary depending on the child’s susceptibility to these exposures. Infants are predisposed to reject bitter tastes (e.g. green vegetables) [24, 25], but early preferences are modifiable through the introduction of vegetables early in complementary feeding [26–28], repeated exposures [25, 27, 29–33], and increased dietary variety [27, 31, 19, 33]. As such, exposure to healthful foods during the first two years of life, while taste preferences and food acceptance are developing, may be more critical for infants with stronger food avoidance traits. Conversely, infants innately prefer sweet tastes [34, 35] and begin to show preferences for salty tastes at approximately 4–6 months, leading to rapid acceptance of discretionary foods [34–37]. Consequently, early and frequent exposure to these foods may result in children subsequently preferring and seeking these foods, especially among those with stronger food approach traits. However, the interaction of infant appetitive traits with dietary exposures has not previously been examined.

The purpose of this study was to investigate the association of infant food exposures with early childhood diet quality, and the moderation of these associations by appetitive traits. We hypothesized that greater infant exposure to fruit and vegetables, and lower exposure to discretionary foods, would be associated with higher diet quality in early childhood. Further, we hypothesized that the association of infant fruit and vegetable exposure with child diet quality would be stronger among those with higher food avoidance traits, and that the association of infant discretionary food exposure with child diet quality would be stronger among those with higher food approach traits.

## Methods

### Design and participants

Data are from the Pregnancy Eating Attributes Study (PEAS) and Sprouts follow-up study, a prospective observational cohort followed from the first trimester of pregnancy through the offspring’s early childhood [38]. PEAS participants were recruited from two university-based obstetrics clinics in Chapel Hill, North Carolina from November 2014 through October 2016. Pregnant persons ≤ 12 weeks gestation were eligible if they met the following criteria: age ≥ 18 and < 45; BMI ≥ 18.5 kg/m^2^; able to complete self-report assessments in English; access to Internet with email; plan to deliver at the University of North Carolina (UNC) Women’s Hospital; plan to remain in the geographical vicinity of the clinical site for one year following delivery; and willingness to undergo study procedures and provide informed consent for self and offspring participation. Exclusion criteria included pre-existing diabetes; multiple pregnancy; participant-reported eating disorder; any medical condition contraindicating participation in the study such as chronic illnesses or use of medication that could affect diet or weight; and psychosocial condition contraindicating participation in the study. The study sample size was based on the primary research question examining associations of reward-related eating and the home food environment with diet and weight outcomes [38]. Participants who remained in PEAS through the one-year follow-up and who consented for future contact were eligible for enrollment in Sprouts at child age 3.5 years. Sprouts exclusion criteria were child diagnosis of neurocognitive disability or attention deficit/hyperactivity disorder. Sprouts enrollment was conducted from February 2019 to December 2020.

### Procedures

PEAS study staff screened the electronic clinical appointments and medical records database to identify potential participants with upcoming clinic visits. During the clinic visit, staff verified eligibility and obtained written informed consent. PEAS study visits were conducted prenatally at < 12 weeks gestation, 16–22 weeks gestation, and 28–32 weeks gestation, and postpartum at 4–6 weeks, 6 months, and 12 months. Self-report measures were completed online within each study visit window.

In anticipation of the Sprouts follow-up study, a brief online assessment was added at child age 2 years. Research staff contacted PEAS participants by email approximately one week prior to the child’s second birthday and invited them to participate in an online survey of their child’s current eating habits. Written information regarding the assessment was provided by email, including documentation that completion of the survey indicated consent for use of the data.

PEAS participants were invited by email to participate in Sprouts; those indicating willingness to participate were contacted by telephone to schedule the initial in-person assessment at child age 3.5 years. Parents provided written informed consent for their and their child’s participation. Due to public health precautions necessitated by the COVID-19 pandemic, recruitment and assessments were completed remotely beginning in March 2020; these parents received the consent form by email, were given the opportunity to ask questions, and confirmed consent via an online form prior to enrollment. Subsequently, assessments at child age 5 years were all completed remotely, rather than in-person as originally planned. Study procedures for PEAS (including the age 2 years assessment) and Sprouts were approved by the University of North Carolina Institutional Review Board.

### Measures

*Infant food exposures.* Duration of any breastfeeding was measured using questions from the Infant Feeding Practices Study [39] at postpartum visits. Beginning at the 6-month visit, parents completed food frequency questionnaires based on validated assessments from the Infant Feeding Practices Study [39] and the Growing Leaps and Bounds study [40]. Items queried the age at which each food was introduced and the current frequency of intake. Responses across assessments were used to determine the age at introduction to selected food groups. At child age 2 years, parents completed a validated food frequency questionnaire developed for infants and toddlers [41]. Exposures examined included the age (in months) at introduction to fruit and vegetables, age at introduction to discretionary foods, and the frequency (times per day) of consuming each food group at ages 1 year and 2 years.

*Infant appetitive traits*. At 6 months postpartum, parents completed the Baby Eating Behavior Questionnaire, which measures appetitive traits during the period of exclusive milk-feeding. The measure includes two food approach subscales – food responsiveness (6 items, *α* = 0.80; e.g., “if given the chance my baby would always be feeding”) and enjoyment of food (4 items; *α* = 0.75; e.g., “my baby enjoyed feeding time”) and two food avoidance subscales – satiety responsiveness (3 items; *α* = 0.39; e.g., “my baby got full up easily”) and slowness in eating (4 items; *α* = 0.62; e.g., “my baby fed slowly”). Responses are provided on a 5-point Likert scale (1 = “never,” 5 = “always”). Items are reverse-scored when appropriate and subscales are scored as the mean of items, with a higher score indicate stronger endorsement of that trait. The measure has previously shown adequate psychometric properties including good internal consistency and a factor structure consistent with the constructs of interest [42].

*Child diet quality*. Parents completed two non-consecutive interviewer-administered 24-h dietary recalls associated with each Sprouts study visit, reporting all food and beverages consumed by the child the previous day, including details such as preparation method, portion size, brand names, and additions. Trained research staff administered the recall interviews using the multiple pass method and the Nutrition Data System for Research software version 2020 (Nutrition Coordinating Center, University of Minnesota, Minneapolis, Minnesota). Quality assurance checks were conducted by the UNC Nutrition Obesity Research Center – Diet and Physical Activity Core, including review of individual food entries above specified energy or weight thresholds and review of records above or below specified threshold energy intakes. Dietary intake data were used to calculate the Healthy Eating Index-2020 (HEI), an a priori indicator of diet quality that indicates conformance to the 2020 US Dietary Guidelines for Americans [43], at ages 3.5 and 5 years. The HEI was calculated across both diet records at each age using the simple scoring method, which sums 13 component scores, including 9 “adequacy components” (Total Fruit, Whole Fruit, Total Vegetables, Greens and Beans, Whole Grains, Dairy, Total Protein, Seafood and Plant Proteins, and Fatty Acids) and 4 “moderation components” (Refined Grains, Sodium, Added Sugars, and Saturated Fats), which are calculated on a per-1000 kcal, percent of energy, or intake ratio basis[44, 45]. All subscales are scored such that a higher score indicates greater adherence to dietary guidelines. The maximum score of 100 reflects adherence to all dietary guidelines. Dietary component scores representing adherence to adequacy components and to moderation components were also calculated.

*Demographic characteristics*. Parents reported sociodemographic information including household composition, income, education, marital status, race/ethnicity, and participation in Special Supplemental Nutrition Program for Women, Infants, and Children (WIC) at the initial PEAS visit. Income-to-poverty ratio was calculated based on family income and household size [46]; higher values indicate greater income relative to the poverty threshold. Parent and child ages and parent parity were obtained from electronic medical records.

### Analyses

**Fig. 1:**
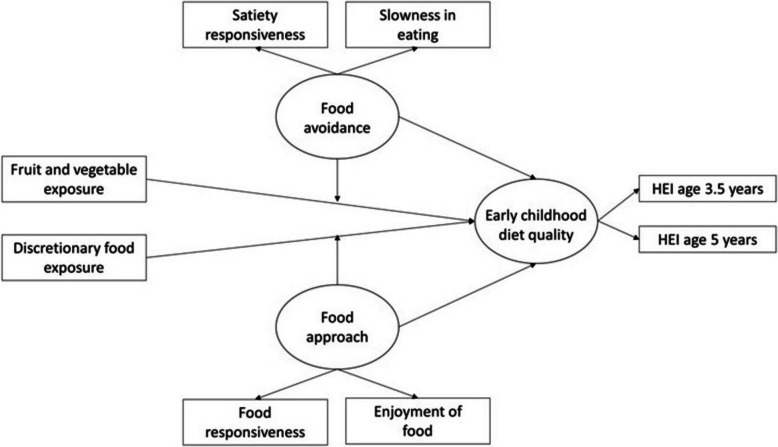
Structural equation model representing hypothesized associations of infant food exposures and appetitive traits with early childhood diet quality

Structural equation modeling using the lavaan package in R was used to examine associations of infant food exposures and their interactions with appetitive traits on early childhood diet quality. Three separate models examined associations of age at introduction to the two food groups and intake frequency of these food groups at ages 1 year and 2 years with early childhood diet quality. Each model used the same structure, differing only in the exposures tested (Fig. 1). Early childhood diet quality was modeled as a latent variable, indicated by the HEI at 3.5 and 5 years of age. Food avoidance was modeled as a latent variable indicated by satiety responsiveness and slowness in eating. Food approach was modeled as a latent variable indicated by food responsiveness and enjoyment of food. Direct effects of the food exposures and appetitive traits on child diet quality were tested. Analyses employed a two-step modeling process to test interaction effects [47, 48]. In the first step, the measurement model for the latent variables was estimated, and factor scores for food avoidance and food approach traits were predicted for each participant. These scores were then used to create interaction terms with the food exposure variables. A two-stage maximum likelihood procedure was used to handle missing data. Participant characteristics hypothesized to be associated with the exposures and outcomes were evaluated for inclusion as covariates, including income, education, WIC participation, and duration of breastfeeding. Income, education, and duration of breastfeeding were associated with at least one exposure and one outcome, and so were included as covariates in the models. Secondary analyses employed the same models as above, but with either the HEI-adequacy component score or the HEI-moderation component score as the outcome.

## Results

Of 458 participants enrolled in PEAS, 321 were retained through one-year postpartum, 280 provided data at child age 2 years, 162 were enrolled in Sprouts at child age 3.5 years (132 in-person and 30 remotely), and 132 provided remote data at child age 5 years (113 who had completed the age 3.5-year assessment and 19 additional participants who had missed the age 3.5-year assessment). Characteristics of PEAS participants who were retained through one-year postpartum and those participating in either of the first two waves of Sprouts are summarized in Table 1. Sample characteristics of those enrolling in Sprouts were similar to those of the retained PEAS sample, with more than 90% married, nearly a quarter with a bachelor's degree or higher, and approximately 70% non-Hispanic White.

**Table 1:** Sample characteristics of Pregnancy Eating Attributes Study (PEAS) and Sprouts participants

	PEAS (*n* = 321)*	Sprouts (*n* = 184)*
	**Mean ± SD or N (%)**	**Mean ± SD or N (%)**
Age at baseline (years)	30.7 ± 4.5	31.0 ± 4.3
Household poverty-income ratio	4.0 ± 2.0	4.1 ± 1.9
Marital status		
Married/living with partner	271 (92.1)	162 (92.6)
Divorced/widowed/separated/single	26 (7.9)	13 (7.4)
Education		
High school graduate or less	24 (15.0)	9 (5.1)
Some college or associate’s degree	53 (16.5)	37 (21.1)
Bachelor’s degree	88 (27.4)	51 (29.1)
Master’s/advanced degree	132 (41.1)	78 (44.6)
Race/ethnicity		
White, non-Hispanic	213 (68.2)	128 (69.9)
Black, non-Hispanic	50 (15.6)	24 (13.1)
Asian, non-Hispanic	16 (5.0)	7 (3.8)
Multi-race or other	11 (3.4)	6 (3.3)
Hispanic	25 (7.8)	18 (9.8)
Participation in Special Supplemental Nutrition Program for Women, Infants, and Children	38 (12.8)	23 (13.2)
Parity		
Nulliparous	136 (42.4)	76 (41.3)
Parous	185 (57.6)	108 (58.7)
Duration of any breastfeeding (months)	8.9 ± 4.5	9.3 ± 4.4

*Analytic sample includes PEAS participants retained through 12-months postpartum and Sprouts participants at age 3.5 and/or 5 years follow-up. Missing data on income-to-poverty ratio for 27 PEAS and 11 Sprouts participants, marital status and education for 24 PEAS and 9 Sprouts participants, race/ethnicity for 6 PEAS and 1 Sprouts participants, participation in Women Infants Children for 24 PEAS and 10 Sprouts participants, and duration of breastfeeding for 23 PEAS and 7 Sprouts participants

Descriptive information on the exposures and outcomes is provided in Table 2. Mean age at introduction to fruit and vegetables was 5.7 months, while mean age at introduction to discretionary foods was 7.8 months. Mean intake of fruit and vegetables at ages 1 and 2 years was 4.7 and 5.5 times/day, respectively. Intake of discretionary foods was 1.7 and 3.1 times/day, respectively, at ages 1 and 2 years. HEI at both ages 3.5 and 5 years was approximately 59.

**Table 2:** Descriptive statistics summarizing infant food exposures, infant appetitive traits, and early childhood diet quality

	**Mean ± SD**
Age at introduction to food groups (months)	
Fruits and vegetables	5.66 ± 1.62
Discretionary foods	7.78 ± 1.93
Intake frequency of food groups at age 1 year (times/day)	
Fruits and vegetables	4.68 ± 2.52
Discretionary foods	1.68 ± 1.73
Intake frequency of food groups at age 2 years (times/day)	
Fruits and vegetables	5.52 ± 4.87
Discretionary foods	3.12 ± 3.82
Infant appetitive traits	
Satiety responsiveness	2.24 ± 0.59
Slowness in eating	2.40 ± 0.62
Food responsiveness	2.23 ± 0.65
Enjoyment of food	4.49 ± 0.47
Healthy Eating Index 2020 age 3.5 years	
Total score	59.78 ± 12.14
Adequacy components	34.51 ± 8.17
Moderation components	25.27 ± 5.53
Healthy Eating Index 2020 age 5 years	
Total score	58.89 ± 13.36
Adequacy components	34.99 ± 8.77
Moderation components	23.90 ± 6.08

**Fig. 2:**
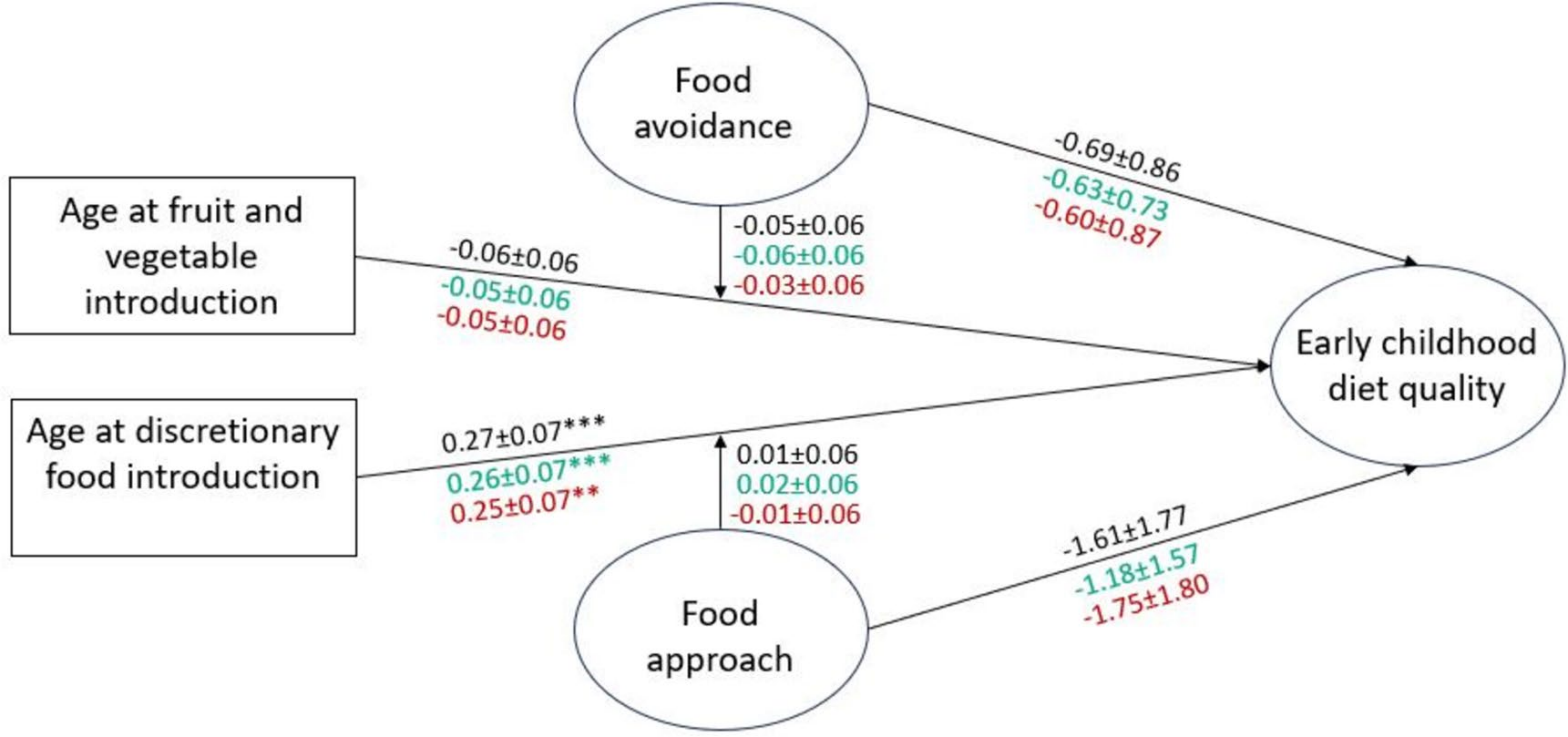
Associations of infant age at introduction to fruits/vegetables and discretionary foods and appetitive traits with early childhood diet quality Coefficients for the model with total diet quality as the outcome are shown in black (CFI 0.84, TLI 0.93, RMSEA 0.05, SRMR 0.08). Coefficients for the model with adequacy components as the outcome are shown in green (CFI 0.94, TLI 0.93, RMSEA 0.05, SRMR 0.08). Coefficients for the model with moderation components as the outcome are shown in red (CFI 0.94, TLI 0.92, RMSEA 0.05, SRMR 0.08). * indicates *p* < 0.05, ** indicates *p* < 0.01, *** indicates *p* < 0.001

**Fig. 3:**
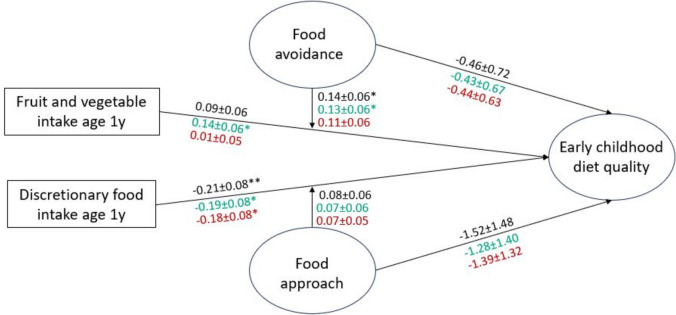
Associations of intake frequency of fruits/vegetables and discretionary foods at age 1 year and appetitive traits with early childhood diet quality Coefficients for the model with total diet quality as the outcome are shown in black (CFI 0.96, TLI 0.96, RMSEA 0.04, SRMR 0.08). Coefficients for the model with adequacy components as the outcome are shown in green (CFI 0.96, TLI 0.95, RMSEA 0.04, SRMR 0.08). Coefficients for the model with moderation components as the outcome are shown in red (CFI 0.95, TLI 0.94, RMSEA 0.04, SRMR 0.08). * indicates *p* < 0.05, ** indicates *p* < 0.01, *** indicates *p* < 0.001.

**Fig. 4:**
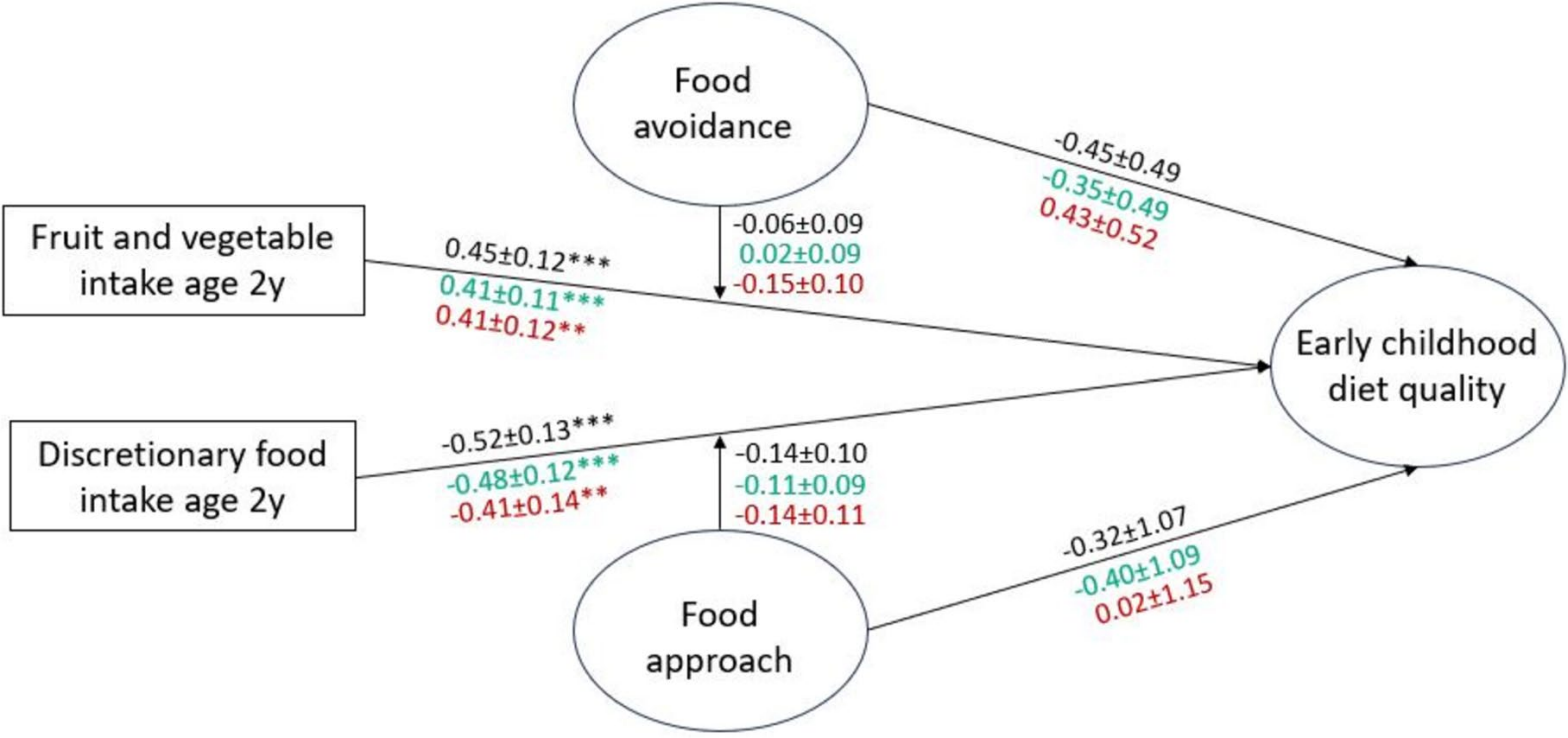
Associations of intake frequency of fruits/vegetables and discretionary foods at age 2 years and appetitive traits with early childhood diet quality Coefficients for the model with total diet quality as the outcome are shown in black (CFI 0.91, TLI 0.89, RMSEA 0.06, SRMR 0.10). Coefficients for the model with adequacy components as the outcome are shown in green (CFI 0.90, TLI 0.88, RMSEA 0.06, SRMR 0.10). Coefficients for the model with moderation components as the outcome are shown in red (CFI 0.90, TLI 0.88, RMSEA 0.06, SRMR 0.10). * indicates *p* < 0.05, ** indicates *p* < 0.01, *** indicates *p* < 0.001

Structural equation models examining associations of infant food exposures with early childhood diet quality, overall and for adequacy and moderation components, are shown in Figs. 2–4. Age at introduction to fruit and vegetables was not associated with overall early childhood diet quality. Later age at introduction to discretionary foods was associated with better diet quality in early childhood; this association was not moderated by infant food approach traits. Associations of later age at introduction to discretionary foods with adequacy and moderation components of early childhood diet quality were of similar magnitude (Fig. 2).

The main effect of the frequency of fruit and vegetable intake at age 1 year on overall early childhood diet quality was not significant; however, a significant interaction term indicated that the association was stronger among infants with greater food avoidance traits. In models examining adequacy and moderation components separately, fruit and vegetable intake at age 1 year was positively associated with early childhood adequacy components, and this association was stronger among infants higher in food avoidance traits. Fruit and vegetable intake at age 1 year was not associated with early childhood moderation components. Discretionary food intake at age 1 year was inversely associated with overall early childhood diet quality, and there was no interaction with food approach traits. Further, discretionary food intake at age 1 year was similarly associated with early childhood adequacy and moderation components (Fig. 3).

Early childhood diet quality was associated positively with fruit and vegetable intake and negatively with discretionary food intake at age 2 years; neither association was moderated by infant appetitive traits. These associations were of similar magnitude in models examining adequacy components and those examining moderation components (Fig. 4).

## Discussion

In this cohort of children followed from birth through age 5 years, exposures to fruit and vegetables and to discretionary foods in the first two years of life were prospectively associated with early childhood diet quality. These associations may be driven both by parent values and preferences when choosing foods to feed their child as well as by child preferences and behaviors developed in response to previous food exposures. The extent to which the associations observed herein were attributable to each of these aspects cannot be determined from this study. However, observational and experimental research demonstrating associations of infant food exposures with subsequent acceptance, preferences, and intake [19, 28, 33, 49, 50] suggests a mechanism by which infant food exposures may impact long-term diet quality.

The association of exposure to fruit and vegetables with child diet quality varied across exposure assessments and by infant appetitive traits. While fruit and vegetable intake at two years of age was associated with subsequent diet quality regardless of appetitive traits, the association of intake at one year of age with child diet quality was moderated by food avoidance traits, suggesting a possible critical window of exposure to fruits and vegetables for children with greater food avoidance. The absence of an association of age at introduction to fruits and vegetables with childhood diet quality suggests that regular exposure to fruits and vegetables across development may be more important than the initial timing of introduction. Alternatively, since more than three-quarters of the sample introduced fruits and vegetables by six months of age, there may have been insufficient variance to observe an association, if one exists.

Discretionary foods were introduced early and fed regularly (on average, nearly twice a day at one year of age and more than three times a day at two years of age) in this relatively highly educated sample. Exposure to discretionary foods in the first two years of life (including age at introduction, intake at age one year, and intake at age two years) was consistently associated with lower childhood diet quality. These associations were observed for both adequacy and moderation components, suggesting that exposure to discretionary foods in infancy may reduce children’s subsequent acceptance of healthful foods. Additionally, infant appetitive traits did not modify associations of infant exposure to discretionary foods with early childhood diet quality. Appetitive traits are believed to reflect individual susceptibility to an obesogenic food environment [51, 52]. However, the consistent direct association of early exposure to discretionary food with subsequent worse diet quality across all analyses, along with the absence of moderation by appetitive traits, suggests that children are susceptible to discretionary foods across the range of stronger and weaker food approach traits.

Consistent associations of discretionary food exposure in the first two years of life with child diet quality suggest the need for greater efforts to reduce exposure to discretionary foods during this developmental period. Current dietary guidelines recommend that parents avoid feeding foods with added sugars in the first two years of life [53]. Yet parents have reported perceiving such recommendations as unrealistic due to the prevalence of these foods in the home and their intake by other members of the family [54, 55]. Parents report feeding discretionary foods not only for their convenience and acceptability, but also to entertain their infant and to show affection [55–57]. As such, public health approaches to promote healthful infant feeding may require improving both the household and the broader food environment and reshaping social norms regarding appropriate foods for infants.

Findings from this study are subject to limitations imposed by the study design and sample. The sample was recruited from a single geographic area that is more highly educated and less diverse than the US population, which may limit generalizability. Additionally, parent report of infant feeding exposures may be subject to social desirability bias, and report of age at introduction to foods may also be subject to recall bias, both of which would likely increase type II error. Despite validation of the Baby Eating Behaviors Questionnaire across numerous samples, the low internal consistency of the 3-item satiety responsiveness subscale suggests this measure may not represent a distinct construct in this sample. However, fit indices for the structural equation models, in which the latent variable of food avoidance traits was indicated by both satiety responsiveness and slowness in eating, were adequate. While there was considerable attrition from PEAS to Sprouts, partly attributable to the COVID-19 pandemic, the sociodemographic characteristics of the Sprouts participants were similar to those of the original sample. The prospective study design, use of validated measures including 24-h recalls assessing early childhood dietary intake, and consideration of sociodemographic covariates all support the internal validity of the findings.

## Conclusions

Findings from this study contribute to a growing body of evidence indicating that dietary exposures in infancy may play a crucial role in shaping long-term diet quality. While the interaction of fruit and vegetable intake at age 1 year with appetitive traits suggests a possible sensitive period for infants high in food avoidance traits, most associations were not modified by appetitive traits, indicating the importance of healthful infant exposures for all children. Exposure to discretionary food in the first two years of life was common and was consistently associated with lower diet quality in early childhood – both adequacy and moderation components – regardless of the strength of appetitive traits. Findings suggest that improving child diet quality may require stronger efforts to limit exposure to discretionary foods in infancy.

## Data Availability

Data described in the manuscript, code book, and analytic code will be made available upon request pending approval of a data use agreement. Following publication of the study objectives, de-identified data will be shared in the NICHD Data and Specimen Hub.

## Abbreviations

HEI: Healthy Eating Index
PEAS: Pregnancy Eating Attributes Study
UNC: University of North Carolina
